# The association of pain phenotype and providing caregiving with depressive symptom trajectory for older adults: a longitudinal analysis using the health and retirement study

**DOI:** 10.1186/s12877-025-05926-5

**Published:** 2025-04-23

**Authors:** Shuqing Zhao, Longhui Chen, Yanfang Duan, Haochen Wang, Chongzhong Liu, Xiaoyun Wang

**Affiliations:** 1 Department of Hepatobiliary and Pancreatic Surgery, The Second Hospital, Cheeloo College of Medicine, Shandong University, Jinan, 250033 China; 2 Cheeloo College of Medicine, Shandong University, Jinan, 250012 China; 3 Director of Nursing, Nursing Department, The Second Hospital, Cheeloo College of Medicine, Shandong University, No.247 Beiyuan Avenue, Tianqiao District, Jinan, 250033 Shandong P. R. China

**Keywords:** Pain, Phenotype, Depressive, Caregiving, Trajectory

## Abstract

**Background:**

This study aims to analyze the association between different pain phenotypes and depressive symptom trajectory among community-dwelling older adults, and whether and how providing caregiving render older adults with pain more prone to a deteriorating depressive trajectory.

**Methods:**

Data were derived from the Health and Retirement Study between 2008 and 2020. Older adults were classified into different pain phenotypes leveraging four pain characteristics at baseline (i.e. 2008) using latent class analysis, including pain intensity, pain interference, pain location, and pain persistence. Caregiving status was collected at baseline using helper lists. Depressive symptoms were measured biennially from 2008 to 2020. Linear mixed models were constructed to explore the independent and interacted effects of pain phenotypes and providing caregiving on depressive symptom trajectory.

**Results:**

Among 8486 participants aged 60 years old or over (58.8% females, and mean age of 74.28), four pain phenotypes were identified: Severe-persistent pain group (15.0%), Moderate pain group (17.3%), Back pain group (7.0%), and Pain-free group (60.7%). Compared to the Pain-free group, other three pain subtypes exhibited higher baseline depression symptoms with a gradient trend. Older adults with Severe-persistent pain had a significantly slower rate of depression symptom increase. Caregiving did not moderate the impact of any pain subtype on baseline depression symptoms, but it significantly reduced the rate of increase in depression symptoms for both Severe-persistent pain group and Back pain group.

**Conclusions:**

Pain phenotype-informed depression services should be delivered. Promoting the caregiving benefit finding for the elderly would contribute to the remission of depressive symptom.

**Supplementary Information:**

The online version contains supplementary material available at 10.1186/s12877-025-05926-5.

## Introduction

Recognizing geriatric syndromes, which are multifactorial and encompass chronic physical, psychological, and social conditions, is crucial for clinical geriatric nursing to mitigate their impact on daily life [1]. Among these, chronic pain is a prevalent syndrome affecting physical function, with rates ranging from 27.6 to 48.5% in community-dwelling older adults [2, 3]. Chronic pain is a well-established predictor of negative outcomes, including falls, reduced quality of life, disability, increased healthcare costs, and mortality [4]. In addition to its physical effects, chronic pain often leads to emotional distress, increasing vulnerability to psychological conditions, particularly depression. Studies show that chronic pain heightens susceptibility to depression both cross-sectionally and longitudinally [5, 6]. Mendelian randomization studies further support a causal relationship, where pain traits predict depression rather than the reverse [7].

Despite this association, the degree of depression experienced by individuals with chronic pain varies significantly. Recent research highlights that specific pain characteristics, such as pain intensity, persistence, location, and interference, differentially impact depression. For instance, the ActiFE Ulm cohort study identified multisite pain, severity, and frequency as strong predictors of late-life depression, with pain severity remaining significant after adjusting for confounders [8]. In contrast, the MOBILIZE-Boston study found multisite pain had a stronger association with depression than pain severity [9]. Pain interference has also been recognized as a key determinant of pain-related depression. Notably, individuals often experience multiple pain characteristics simultaneously, which interact to influence emotional responses. Focusing on a single pain characteristic may thus introduce bias, underscoring the need for a multidimensional approach to better characterize pain phenotypes and their psychological effects in older adults.

Phenotypes, defined as clusters of observable traits that distinguish subgroups within a population, have been proposed as a framework for tailoring care models for individuals with pain [10, 11]. A recent pain phenotyping study applied unsupervised latent class analysis to explore the existence of distinct pain phenotypes, utilizing indicators of pain severity, interference, count, duration and pain-related worry as input variables for extracting phenotypes. This study identified three pain phenotypes—“low,” “moderate,” and “high”—with varying prevalence of mental disorders [12]. Joint latent class analyses of pain and psychological traits further showed that different pain intensities correspond to distinct psychological states, without a clear dose-response relationship between pain and depression [13, 14]. While individuals with more severe pain are generally more likely to report anxiety and depression [15, 16], most studies have focused on cross-sectional data from specific diseases or younger populations. As a result, the longitudinal impact of pain phenotypes on depressive symptom, particularly in older adults, remains under-explored. Notably, most existing studies analyzed the relationship between individual pain characteristics and the onset and progression of depression, with limited exploration of their association with depression symptom trajectories. It remains unclear whether and how different pain phenotypes, derived from pain characteristics, influence depression symptom trajectories in older adults. This gap in knowledge limits the generalizability and clinical applicability of existing findings, highlighting the need for longitudinal research that explores the long-term effects of pain phenotypes on mental health across the lifespan.

Biopsychosocial models of clinical pain acknowledge the complex intersection of pain experiences with social roles [17]. Caregiving, a common role among older adults aging in place, probably adds further complexity by influencing pain-related experiences. Given that caregiving has been associated with variations in physical and mental health, including an increased risk of both severe pain and depressive symptoms among caregivers [18, 19], it is essential to explore how this role might impact the relationship between pain phenotypes and emotional distress. While some studies suggest that pain intensity predicts depression in older caregivers [20], others show that caregiving may not always exacerbate emotional distress, with some caregivers reporting less pain [21]. In that case, depression as the attachment of an emotionally negative character to pain sensations would not be inferred. Therefore, it remains unknown whether and how providing caregiving affects the development of negative emotions (e.g. depression) stemming from pain perception in older adults. It is also unclear whether providing caregiving influences the change of depression symptom levels across different pain phenotypes.

This study aims to (1) identify potential pain phenotypes among community-dwelling older adults, (2) examine the association between different pain phenotypes and depressive symptom trajectories, and (3) explore whether and how caregiving influences this relationship. Addressing these questions will help determine if caregiving increases older adults’ susceptibility to the negative psychological effects of pain and predict potential depressive changes in older caregivers with varying pain phenotypes. Additionally, given the limited interventions targeting physical pain in caregivers and evidence suggesting that pain relief alone may not reduce depression, this research could inform the development of phenotype-specific interventions for older caregivers that address both physical pain and psychological well-being.

## Methods

### Participants and sample

This longitudinal study utilized data from the Health and Retirement Study (HRS), a population-based cohort of community-dwelling older adults in the United States. This study oversampled minority populations, such as Black and Hispanic individuals. Participants undergo detailed in-person or telephone interviews approximately every two years from cohort entry until death or dropout [22]. The biennial core interviews collect demographic, physical, and psychosocial data. For this study, data from Section G: Functional Limitations and Helpers were merged with participants’ IDs to include caregiving-related variables. The main dataset was derived from the HRS core interviews conducted from wave 9 (2008) to wave 15 (2020). Wave 9 was chosen as the baseline because it is the first wave to collect comprehensive pain data following the integration of the HRS 2004 Early Baby Boomers cohort. Before Wave 9, the HRS collected data on pain characteristics including the presence of pain, pain intensity, and pain-related activity limitations, without information on pain location, specifically back pain. The collection of data on back pain began in Wave 9.

**Fig. 1:**
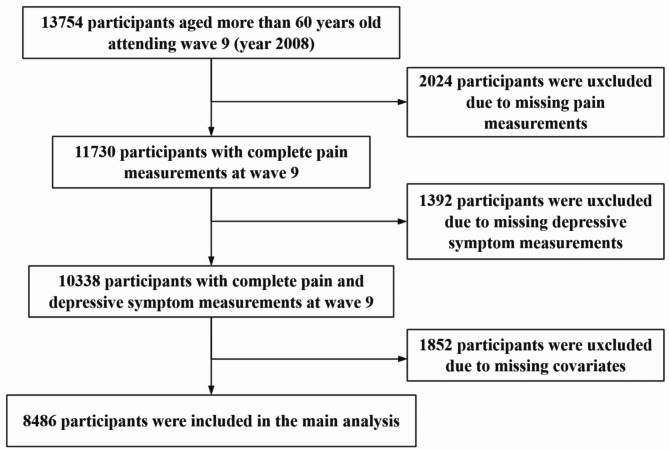
Flowchart of participants

Participants were included if they (1) completed the HRS wave 9 interview and (2) were aged at least 60 years old at baseline. Of the remaining 13,754 participants, some cases were excluded due to they had missing information on key pain characteristics, such as pain persistence, intensity, activity interference, and location, as well as on depressive assessment at baseline. We also excluded individuals with missing data on covariates to reach a complete-case analysis, which yielded a final sample size of 8486 participants (Fig. 1).

All participants in the HRS give verbal informed consent for their participation in the study; HRS data collection is approved by the Health Sciences and Behavioral Sciences institutional review board at the University of Michigan. All analyses used publicly available, non-restricted data collected through the Health and Retirement Survey and did not require IRB/human subject review.

### Measures

#### Depressive symptom

Mental health is conceptualized as depressive symptom, which are measured using an 8-item version of Center for Epidemiological Studies-Depression scale (CES-D8). The scale is an additive score of dichotomous “yes/no” responses to the following statements: was depressed, everything was an effort, sleep was restless, was happy, felt lonely, enjoyed life, felt sad, and could not get going, ranging from 0 (no symptoms) to 8 (all symptoms reported) [23]. Depressive symptom assessed from wave 9 to wave 15 were used in the analysis.

### Pain characteristics

Four key pain characteristics were collected to generate pain phenotypes: pain persistence, pain intensity, activity interference, and pain location [24]. These characteristics were measured at baseline through four pain-related questions. According to the answer to the question “Are you often troubled with pain?“, pain persistence was categorized into three groups. Participants reporting frequent pain at baseline and in subsequent interviews were classified as having persistent pain; those reporting pain only at baseline, with no pain in previous or subsequent interviews, were classified as having single-occurrence pain; and those reporting no pain in any wave were classified as pain-free. According to answers to the question “How bad is the pain most of the time: mild, moderate, or severe?” and the above question, pain intensity was categorized as severe (participants who reported severe pain most of the time), mild/moderate (participants who reported mild or moderate pain most of the time), or pain-free (participants who reported no pain at baseline). According to answers to the question “Does the pain make it difficult to do your usual activities, such as household chores or work?” and the first question, pain interference was categorized as activity-interfering pain (participants who answered yes to this question), non-interfering pain (participants who answered no to this question), or pain-free (participants who reported no pain at baseline). Finally, according to answers to the question “Have you had back pain or problems?”, participants were dichotomized based on the presence or absence of back pain.

### Caregiving

Caregiving status was determined using responses from the Functional Limitation section. Participants reporting difficulties with Activities of Daily Living (ADL) or Instrumental Activities of Daily Living (IADL) were asked whether they received help and to identify their helpers. If a participant was listed as a helper for another person’s ADL or IADL at baseline, they were classified as a caregiver; otherwise, they are classified as a non-caregiver [25].

### Covariates

The models included the following covariates: age, sex, years of education, race, ethnicity, household income, marital status, self-reported health, presence of hypertension, diabetes, heart disease, cancer, and arthritis, smoking status, drinking status, cognitive level (TICS-27 score), and any limitations in ADL. The sample was diverse in terms of demographics. Specifically, in the HRS, participants’ race is categorized as White, Black, and Other, while ethnicity is divided into Hispanic and non-Hispanic. Marital status is classified as married, separated, divorced, widowed, never married, and other. Self-reported health is categorized as excellent, very good, good, fair, and poor. The presence of hypertension is categorized as currently/previously present and never present. The presence of diabetes is categorized as currently/previously present and never present. The presence of heart disease is categorized as currently/previously present and never present. The presence of cancer is categorized as currently/previously present and never present. The presence of arthritis is categorized as currently/previously present and never present. Smoking status is classified as current/past smoker and never smoked. Drinking status is categorized as current/past drinker and never drank. Given that some demographic subgroups had small sample sizes, and to ensure adequate statistical power for each subgroup, we combined certain subgroups when presenting demographic characteristics. For instance, due to the smaller number of participants in the Black, Asian, and Other groups, these categories were combined into the “Other” category for the race variable. Similarly, the groups of separated, divorced, widowed, never married, and other marital statuses were combined into a single “non-married” category for the dichotomous marital status variable. Self-reported health was dichotomized into excellent/very good/good vs. fair/poor. ADL limitations were categorized as binary based on whether the participant reported any dependence on ADL activities (Table 1). All covariates were assessed at baseline based on participant reports. In cases where significant cognitive impairment was detected, the HRS may also obtain data from a proxy respondent (e.g., a family member or caregiver) to provide supplementary information.

**Table 1:** Baseline characteristics of participants

Characteristics	Total sample
Age, years	74.28
Female (%)	4986 (58.8)
Education, years	12.37
Ethnicity (%)	
Hispanic	658 (7.8)
Non-Hispanic	7828 (92.2)
Race (%)	
White	7147 (84.2)
Black	1075 (12.7)
Other^*^	264 (3.1)
Not married (%)	3497 (41.2)
Income, dollars	16475.26
Rated health fair/poor (%)	2344 (27.6)
Having hypertension (%)	5592 (65.9)
Having diabetes (%)	1917 (22.6)
Having cancer (%)	1571(18.5)
Having cardiovascular disease (%)	2568 (30.3)
Having arthritis (%)	5964(70.3)
Ever smoke (%)	4478(52.8)
Ever drink (%)	4031 (47.5)
ADL dependent (%)	504 (5.9)
Cognition level, score	21.94
Baseline depressive symptom, score	1.31
Pain persistence (%)	
Pain-free	4051 (47.7)
Single-occurrence pain	1730 (20.4)
Persistent pain	2705 (31.9)
Pain intensity (%)	
Pain-free	5744 (67.7)
Mild pain	2303 (27.1)
Moderate/severe pain	439 (5.2)
Pain interference (%)	
Pain-free	5744 (67.7)
No Activity-interfering pain	1044 (12.3)
Activity-interfering pain	1698 (20.0)
Having back pain (%)	3089 (36.4)
Providing caregiving (%)	1049 (12.4)

ADL: activity of daily living

*: Race was categorized as White, Black, and Other in the HRS database. Among these, those were identified as “Other” including American Indian, Alaskan Native, Asian, and Pacific Islander

### Statistical analysis

First, Latent Class Analysis (LCA) was conducted to identify pain phenotypes based on the four categorized pain characteristics at baseline. The number of classes was determined through a stepwise model comparison approach (e.g., 1, 2, and 3 classes) using an unconditional model without covariates or distal outcomes. Model fit was assessed using the sample-size adjusted Bayesian Information Criterion (SA-BIC) and entropy, with lower SA-BIC and higher entropy (entropy > 0.70) indicating better model fit. The Lo-Mendell-Rubin and bootstrapped likelihood ratio tests were not used due to the complexity of the data. The optimal number of classes was selected based on fit statistics and class sizes greater than 5%. These analyses were performed in Mplus 7.1.

Descriptive statistics were calculated for the identified pain phenotypes. Means, standard deviations, medians, interquartile ranges, and frequencies were reported, and group differences were tested using t-tests or chi-square tests. We also examined the distribution of pain phenotypes among caregivers and non-caregivers.

To explore the potential effect modification of providing caregiving on the longitudinal association of pain phenotypes and depressive symptoms change, a multivariable linear mixed model was adopted [26]. All covariates were adjusted as fixed effects according to a predefined modeling plan. The primary focus was the interaction between pain subtypes and caregiving status, as well as their interaction with time, to examine how caregiving modifies the baseline (intercept) and longitudinal (slope) changes in depressive symptoms associated with different pain subtypes. Random effects were included for individual slopes and intercepts. A quadratic term for time was tested to explore non-linear associations, and a fully adjusted model was determined through model comparison. Marginal effects and confidence intervals were calculated for the linear predictor at different time points, using the mean values of other covariates. Analyses were performed with Stata 17.0, incorporating HRS respondent weights. Statistical significance was set at *P* < 0.05.

Sensitivity analyses were conducted, including subgroup analyses by gender, age, education, race, marital status, and health status. Multiple imputation was used for missing covariate data, and models were refitted on the imputed datasets. We also examined which components of pain characteristics within each pain phenotype contributed to depression. Additionally, caregiving roles (e.g., spouse, child, relative) were analyzed for their impact on the relationship between pain phenotypes and depressive symptoms. Finally, we tested the mediating role of pain phenotypes in the caregiving-depression relationship by fitting a linear mixed model, first including only caregiving and then adding pain phenotypes to assess changes in caregiving effects.

## Results

### Pain phenotypes from LCA

A total of 8486 participants aged 60 years old or over at baseline were included in this analysis, with a percentage of 58.8% female and mean age of 74.28. LCA was conducted with two up to five classes. Model fit criteria (supplementary material Table 1) suggested that a four-class model represented a good fit to the data (best SA-BIC), also yielding a solid classification accuracy (entropy = 0.861). Increasing the number of classes to five improved only one model fit criterion, SA-BIC, but additional classes became prohibitively small (< 3%).

**Fig. 2:**
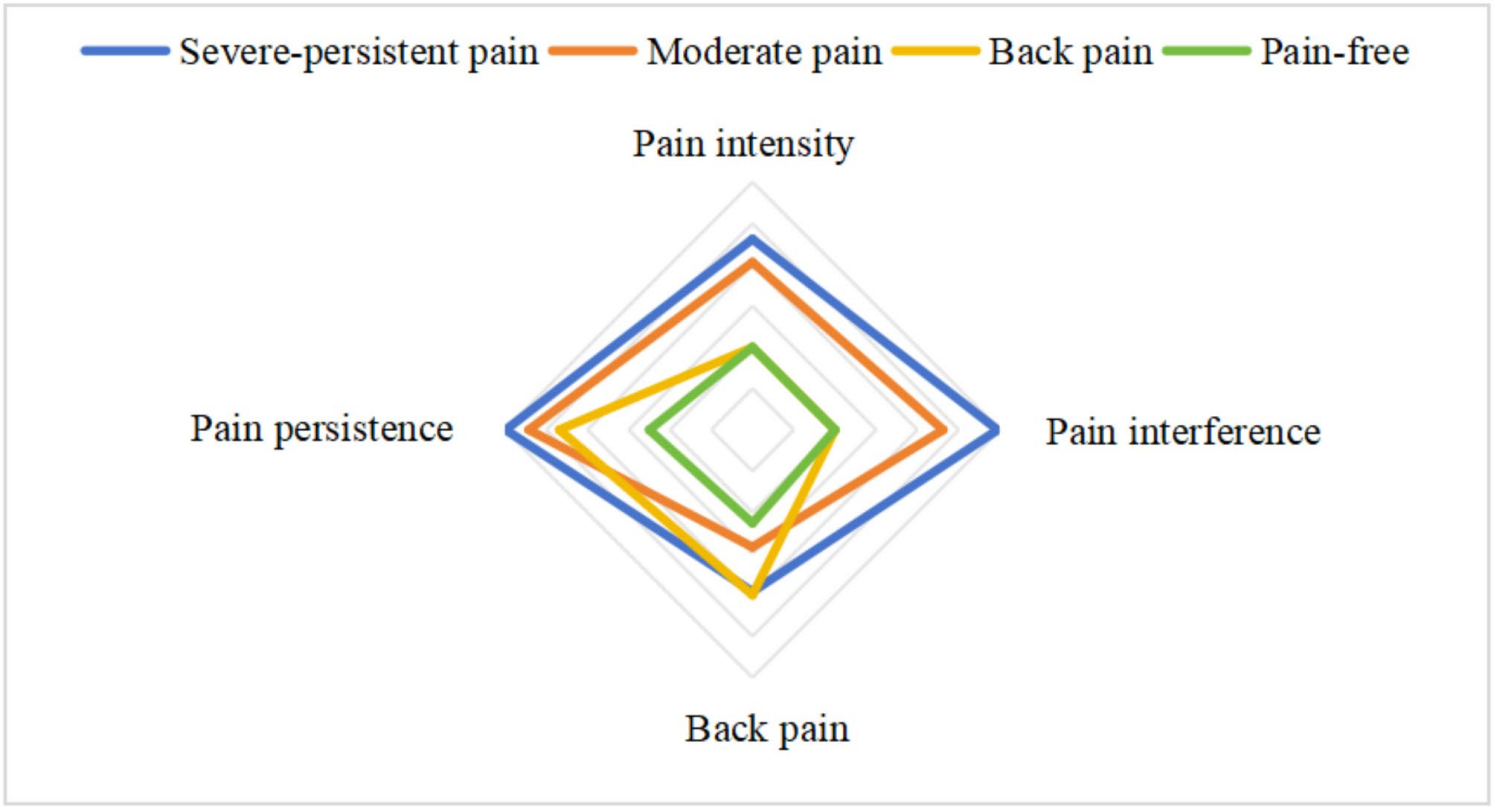
Radar map of pain characteristics distribution of the four pain phenotypes. Footnote: The closer the end of a pain phenotype is to the outside of the rhomboid, the stronger the pain characteristic is

Figure 2 illustrated the chosen 4-class model, which showed a roughly graded intensity (Fig. 2). On average, participants in Class 1 (Severe-persistent pain, 15.0%) had more than 90% probability of reporting activity-interfered pain and persistent pain, as well as prominent back pain; Class 2 (Moderate pain, 17.3%) was characterized by mild pain and is more likely to report persistent pain, although the pain does not necessarily interfere with daily life; Class 3 (Back pain, 7.0%) tended to have back pain issues, although more than 50% of participants reported that the pain occurred in a single wave and did not persist; Class 4 is classified as having the least pain (Pain-free, 60.7%), as over 85% of participants reported ‘none’ for either of the four pain-related characteristics.

### Baseline characteristics description

Baseline characteristics of the 4 pain characteristics classes are summarized in Table 2. Differences between classes were identified for most baseline characteristics, except for age, race, household income, and smoking history. Severe-persistent pain group had more percentage of females, Hispanic ethnicity, self-rated poor health, limited IADL, baseline depressive symptoms, and chronic diseases especially arthritis, while the Pain-free group generally showed a lower frequency in the aforementioned characteristics. Severe-persistent pain group also had less married older adults and younger mean age than other three groups. They had less frequently drunk and received shorter duration of education. Moderate pain group and Back pain group showed significant differences in certain features after applying multiple correction, including pain interference, pain persistency, and having back pain.

**Table 2:** Descriptive characteristics of participants by identified pain phenotype

Characteristics	Severe- persistent pain group (15.0%)	Moderate pain group (17.3%)	Back pain group (7.0%)	Pain-free group (60.7%)	F/c^2^	*P* value
Age, years	74.02	74.07	74.56	74.38	1.81	0.143
Female (%)	909 (71.6)	94 (61.4)	371 (62.4)	2802 (54.4)	133.56	< 0.001
Education, years	11.77	12.28	12.20	12.55	2028	< 0.001
Ethnicity (%)					13.75	0.030
Hispanic	121 (9.5)	133 (9.0)	37 (6.2)	367 (7.1)		
Non-Hispanic	1149 (90.5)	1339 (91.0)	558 (93.8)	4782 (92.9)		
Race (%)					2.22	0.532
White	1077 (84.8)	1254 (85.2)	503 (84.5)	4313 (83.8)		
Other^*^	193 (15.2)	218 (14.8)	92 (15.5)	836 (16.2)		
Not married (%)	615 (48.4)	600 (40.8)	253 (42.5)	2029 (39.4)	34.75	< 0.001
Income, dollars	11490.34	13933.85	13577.26	16475.26	2.07	0.101
Rated health fair/poor (%)	766 (60.3)	490 (33.3)	185 (31.1)	903 (17.5)	968.12	< 0.001
Having hypertension (%)	968 (76.2)	1010 (68.6)	408 (68.6)	3206 (62.3)	97.19	< 0.001
Having diabetes (%)	366 (28.8)	357 (24.3)	154 (25.9)	1040 (20.2)	51.04	< 0.001
Having cancer (%)	244 (19.2)	294 (20.0)	115 (80.7)	918 (17.8)	4.35	< 0.001
Having cardiovascular disease (%)	523 (41.2)	500 (34.0)	227 (38.2)	1318 (25.6)	151.97	< 0.001
Having arthritis (%)	1211 (95.4)	1251 (85.0)	512 (86.1)	2990 (58.1)	973.10	< 0.001
Ever smoke (%)	669 (52.7)	757 (51.4)	310 (52.1)	2742 (53.3)	1.66	0.646
Ever drink (%)	482 (38.0)	693 (47.1)	277 (46.6)	2579 (50.1)	60.56	< 0.001
ADL dependent (%)	217 (17.1)	98 (6.7)	44 (7.4)	145 (2.8)	376.02	< 0.001
Cognition level, score	21.19	22.18	21.58	22.10	13.35	< 0.001
Baseline depressive symptom, score	2.66	1.56	1.55	0.88	383.40	< 0.001
Pain persistence (%)					7307.23	< 0.001
Pain-free	0 (0)	0 (0)	0 (0)	4051 (78.7)		
Single-occurrence pain	16 (1.3)	422 (28.7)	395 (66.4)	897 (17.4)		
Persistent pain	1254 (98.7)	1050 (71.3)	200 (33.6)	201 (3.9)		
Pain intensity (%)					9739.13	< 0.001
Pain-free	0 (0)	0 (0)	595 (100)	5149 (100)		
Mild pain	874 (68.8)	1429 (97.1)	0 (0)	0 (0)		
Moderate/severe pain	396 (31.2)	43 (2.9)	0 (0)	0 (0)		
Pain interference (%)					12411.46	< 0.001
Pain-free	0 (0)	0 (0)	595 (100)	5149 (100)		
No Activity-interfering pain	32 (2.5)	1012 (68.8)	0 (0)	0 (0)		
Activity-interfering pain	1238 (97.5)	460 (31.3)	0 (0)	0 (0)		
Having back pain (%)	1217 (95.8)	619 (42.1)	595 (100)	658 (12.8)	4238.18	< 0.001
Providing caregiving (%)	364 (28.7)	203 (13.8)	78 (13.1)	404 (7.8)	411.45	< 0.001

ADL: activity of daily living

*: Race was categorized as White, Black, and Other in the HRS database. Due to the smaller number of participants in the Black and Other groups, these categories were combined into the “Other” category for the race variable

Differences in pain characteristics and subtype distribution were further examined under caregiving statuses (sTable 2 in the supplementary material). Older adults providing caregiving consistently exhibited greater pain severity across all measures. Among non-caregivers, 63.8% fell into the Pain-free class. In contrast, caregiving participants were evenly distributed between the Pain-free and Severe-persistent pain classes.

Results of linear mixed models.

**Fig. 3:**
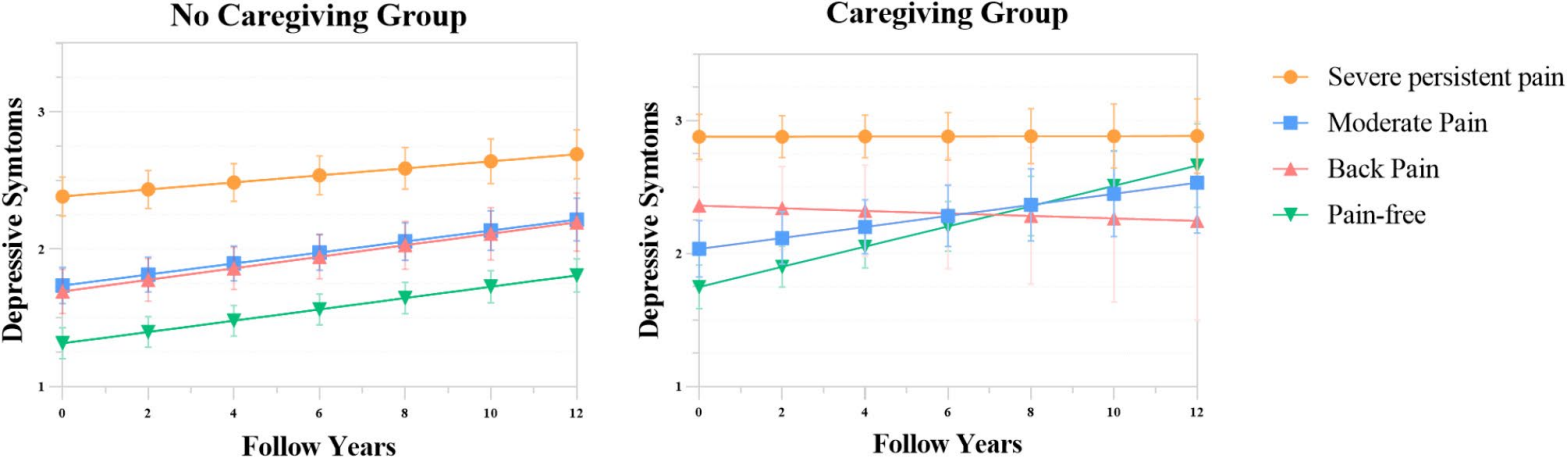
Depressive symptom trajectory of the four pain phenotypes based on the caregiving status

The independent and interacted effects of pain subtypes and providing caregiving on depressive symptom trajectory are presented in Table 3 and plotted in Fig. 3. In the fully adjusted model, the average baseline depression symptom score of the study population was 1.48, which showed a significantly upward trend over the 12-year follow-up period. We selected the Pain-free class as the reference group due to its closest resemblance to a pain-free state and its adequate sample size. Compared to the pain-free group, the other three pain phenotypes exhibited higher baseline depression symptoms, with the Severe-persistent pain group starting off with the highest level of depression symptoms (β = 1.069, 95% CI: 0.962 to 1.175), followed by the Moderate pain group (β = 0.421, 95% CI: 0.331 to 0.510) and the Back pain group (β = 0.378, 95% CI: 0.245–0.508). Older adults with Severe-persistent pain also had a significantly slower rate of depression symptom increase (β=-0.016, 95% CI: -0.029 to -0.002), whereas the Moderate pain group (β=-0.001, 95% CI: -0.013 to 0.010) and the Back pain group (β = 0.001, 95% CI: -0.016 to 0.018) showed similar depression symptom increases over time compared with Pain-free group. In addition, older adults who provided informal caregiving had higher depression symptoms at baseline (β = 0.434, 95% CI: 0.269-0.600), which are roughly comparable to those of the Moderate pain group, as well as faster increase in depression symptom (β = 0.035, 95% CI: 0.007–0.063).

**Table 3:** Linear mixed model of the association between pain phenotype, caregiving status and depressive trajectory, 2008–2020

Characteristics	Coefficient	95% CI	*P* value
Intercept	1.478	1.069 to 1.887	< 0.001
Time, slope	0.041	0.036 to 0.046	< 0.001
Pain phenotype			
Severe persistent pain group	1.069	0.962 to 1.175	< 0.001
Moderate pain group	0.421	0.331 to 0.510	< 0.001
Back pain group	0.378	0.245 to 0.508	< 0.001
Pain-free group	Ref.		
Pain phenotype*time			
Severe persistent pain group*Time	-0.016	-0.029 to -0.002	0.025
Moderate pain group*Time	-0.001	-0.013 to 0.010	0.838
Back pain group*Time	0.001	-0.016 to 0.018	0.931
Pain-free group*Time	Ref.		
Caregiving status			
Providing caregiving	0.434	0.269 to 0.600	< 0.001
Not providing caregiving	Ref.		
Caregiving status*Time			
Providing caregiving*Time	0.035	0.007 to 0.063	< 0.001
Not providing caregiving*Time	Ref.		
Providing caregiving*Pain phenotype			
Providing caregiving*Severe persistent pain group	0.061	-0.173 to 0.295	0.611
Providing caregiving*Moderate pain group	-0.135	-0.397 to 0.127	0.313
Providing caregiving*Back pain group	0.232	-0.150 to 0.615	0.234
Providing caregiving*Pain-free group	Ref.		
Providing caregiving*Pain phenotype*Time			
Providing caregiving*Severe persistent pain group*Time	-0.060	-0.099 to -0.021	0.003
Providing caregiving*Moderate pain group*Time	-0.033	-0.078 to 0.012	0.146
Providing caregiving*Back pain group*Time	-0.086	-0.161 to -0.012	0.024
Providing caregiving*Pain-free group*Time	Ref.		

CI: Credit Limit

Next, we focused on the moderating effect of caregiving on the relationship between pain subtypes and depression trajectories. The results indicated that caregiving did not moderate the impact of any pain phenotype on baseline depression symptoms. However, participants who provided caregiving had significantly slower rate of increase in depression symptoms when they were categorized into Severe-persistent pain (β=-0.060, 95% CI: -0.099 to -0.021) and Back pain (β=-0.086, 95% CI: -0.161 to -0.012) group. For example, caregiving was associated with a reduction in depressive symptom scores on an annual basis by 0.06 points for the Severe-persistent pain group and of 0.09 points for the Back pain group.

### Results of sensitivity analyses

(1) Subgroup analyses indicate that the moderating effect of caregiving on the relationship between pain subtypes and depression is primarily observed among women, older adults under 75 years old, and those with a college education or higher. (2) Random covariate imputation produced consistent results. (3) We explored the pain characteristics driving depression within each pain subtype: in the Severe-persistent pain group, severity and persistence are key factors; in the Moderate pain group, activity interference, presence of back pain, and persistence are significant depression symptoms’ drivers; in the Back pain group, persistence is crucial; and in the Pain-free group, back pain and pain persistence can lead to increased depressive symptoms. These findings not only validate the rationale for pain subtype classification but also highlight the specific pain characteristics that require targeted interventions in different populations. (4) When examining the specific impacts of caregiving roles, depressive symptoms are more severe for all types of caregivers, while depressive symptoms in child caregivers who suffered from pain rise more slowly over time. (5) Comparisons of caregiving’s effects on depression, with and without pain phenotypes, indicated that pain partially mediated caregiving’s negative impact on depressive symptoms (sTable 3–12 in the supplementary materials).

## Discussion

This study identified distinct pain phenotypes among a representative sample of community-dwelling older adults and explored their associations with depressive symptom trajectories, as well as their interactions with caregiving. Four phenotypes emerged: Severe-persistent pain, Moderate pain, Back pain, and Pain-free, each linked to progressively higher baseline depressive symptom severity. Notably, older adults in the Severe-persistent pain group exhibited a slower rate of increase in depressive symptoms over time. While caregivers demonstrated higher baseline depressive symptoms and steeper symptom increases longitudinally, caregiving did not influence baseline depression across pain phenotypes. However, caregivers showed a significantly reduced rate of depressive symptom progression in the Severe-persistent pain and Back pain groups. These results recall a key intention of phenotype-informed care among adults with pain, and provide insight into targeted care to prevent a trajectory into worsening mental state.

By classifying the population based on pain characteristics and intensity, four pain-vulnerable subgroups were identified. A gradient-dependent pattern emerged across these subgroups, consistent with previous studies [12, 27, 28]. For instance, a cross-sectional study on musculoskeletal pain identified three groups: “no pain”, “mild multisite”, and “moderate-severe multisite” pain, in both women and men [28]. Longitudinal studies have also highlighted pain trajectories ranging from mild to severe, with stable, regressing, and progressing patterns [29, 30]. Together with the current findings, these studies could emphasize the key roles of pain severity and persistence in shaping pain trajectories.

Importantly, this study identified a Back pain phenotype, defined by a primary complaint of back pain, despite its mild severity and minimal interference with daily life. This subgroup warrants particular attention, as low back pain is a common precursor to widespread pain, often developing insidiously in the pain drawing studies [31]. Given that 15% of older adults in this study were classified in this group, early interventions such as exercise and preventive measures may substantially benefit this population.

The identified four pain phenotypes have a dose-response relationship with depression levels, indicating that depressive symptoms may progressively worsen from the pain-free group to the severe persistent pain group. This result is consistent with investigations in pain psychology [32], which showed that pain may have its origins not only in damage suffered by nerve fibers, but also in psychogenic background [33, 34]. Additionally, studies on pain phenotypes have shown that mental health outcomes worsen from “mild multisite” to “moderate-severe multisite” pain [28]. While a bidirectional relationship between chronic pain and depression has been documented, recent genetic studies suggest that pain may lead to depression rather than the reverse [7, 35]. By inspecting the effect size of pain phenotypes on depression trajectories, this study deepens our understanding, showing that pain phenotypes do not significantly accelerate depressive symptom progression. In fact, older adults with Severe-persistent pain showed a reduced rate of depressive symptom increase. Integrating these findings with epidemiological and genetic research suggests that older adults with more advanced pain phenotypes may experience an earlier onset of depressive symptoms, but not a faster progression compared to their pain-free counterparts. Furthermore, older adults with severe pain may develop adaptive mechanisms, fostering resilience and aiding mental health recovery [36, 37]. The onset of pain could also likely trigger role adjustment and support-seeking behaviors, facilitating depression recovery throughmay secondary gain mechanisms from somatic expression [38].

The findings regarding caregiving were somewhat unexpected. Both this study and prior research have indicated that elderly caregivers often experience higher levels of pain and depression, along with a greater likelihood of psychological pain [19, 39]. This leads to the assumption that providing caregiving could exacerbate the negative effects of pain on depression. However, the findings of this study suggest that becoming a caregiver may not be associated with the susceptibility of elderly individuals with existing pain to the progression of depression. Moreover, sensitivity analysis revealed that caregiving can affect depression levels through the mediation of pain phenotypes. This suggests that when caring for others leads to physiological changes, such as the onset or exacerbation of pain, it may often impact on mental health as well [40]. These effects may stem from how elderly individuals with pain interpret feedback from significant others [41], which can be either positive and supportive or negative and indifferent, thereby might mitigate the caregiving impact. Future research could examine different aspects of caregiving, such as burden and benefits, to assess how these factors influence the relationship between pain and psychological well-being.

Notably, caregiving was found to be associated with a slower progression of depressive symptoms in older adults with severe persistent pain and back pain. It is important to note that this result does not advocate for older adults with moderate to severe pain to provide caregiving. Rather, the findings suggest that the unexpected yet promising result observed within the caregiver group could prompt further consideration of potential interventions. Specifically, the positive relationship identified indicates that there may be potential to improve the psychological well-being of older caregivers who experience physical pain. Recent research on caregiver benefit discovery [42] suggests that caregiving may promote psychological growth and resilience. Thus, one potential explanation for this finding is that providing caregiving may alleviate pain-related depression by shifting negative cognitive biases toward pain and fostering positive emotional experiences. Intervention studies have emphasized the importance of addressing negative pain perceptions (e.g., pain catastrophizing) and enhancing mindfulness to improve emotional functioning in chronic pain patients [43, 44]. Although negative emotions that arise during caregiving are inevitable, older adults may develop emotional stability and coping mechanisms [45], which could help them adapt to persistent pain, potentially slowing the worsening of depressive symptoms. Additionally, previous reviews have referred to the resilience among individuals with high levels of pain severity [46, 47]. In these studies, individuals with severe pain may exhibit resilient characteristics under the influence of positive trait resources and with fewer pain-related functional impairments, which could result in their reporting a lower emotional burden. Other empirical research also has suggested that severe pain can help maintain psychological well-being over time particularly among female spouses, which may be prompted by increased resilience in caregivers [48]. This transformation of psychological mechanisms may require time, which could be why our analysis only found this reflected in the longitudinal effects on the slope of depression in this study. Further exploration of mechanisms related to the moderating effect of caregiving in association between pain experience and depressive symptom progression is needed.

Our findings suggest that phenotype-informed care may have potential value for adults with pain [12]. Further research is needed to validate the effectiveness of specific treatments and interventions tailored to each pain phenotype group. Based on the identified pain phenotypes, interventions should be framed in a manner that considers both the stage and intensity of psychological interventions, as well as the caregiving status of individuals. For elderly caregivers, a home-based nursing support and cognitive restructuring intervention may be beneficial to promote positive thinking and emotion [49]. Besides, mindfulness therapy based on digital devices may enhance quality of life and emotional resilience for caregivers [50]. Additionally, interventions based on the diathesis-stress model may help foster adaptive personality traits and coping mechanisms [51–53]. These interventions are likely to provide greater benefits when tailored to the caregiving role, helping to alleviate the psychological burden of caregiving while addressing the negative psychological effects of pain. Furthermore, these approaches may be especially beneficial for sensitive subgroups, such as women and child caregivers, as suggested by our sensitivity analyses. However, further research is needed to confirm this claim.

### Strengths and limitations

This study used a data-driven approach to analyze pain characteristics among community-dwelling older adults and examined factors contributing to depression progression within each pain phenotype. It also explored the moderating effect of caregiving, expanding our understanding of caregiving’s impact on physical and psychological health. However, several limitations should be noted. First, due to the complexity of data interpretation, we did not analyze the effect of time-point changes in pain phenotypes on depression, though previous studies suggest that pain phenotype subgroups remain relatively stable over time [29]. Additionally, the time-dependent effects of covariates were not considered. Second, our analysis relied on a complete-case approach, excluding cases with missing data, which may introduce selection bias and limit the generalizability of our findings. Additionally, the pain phenotype variables were constrained by data availability, excluding potentially relevant information, such as specific pain sites, which could provide additional insights into the pain experience. Finally, data on care intensity, duration, and recipients were not collected. These factors may have had an impact on the results, particularly in understanding the broader context in which pain phenotypes and caregiving status interact. This underscores the need for future research that incorporates these factors to enhance the generalizability and depth of the analysis.

## Conclusion

This national cohort study of community-dwelling older adults identified four pain phenotypes, providing a basis for implementing phenotype-informed depression services over time. Caregiving does not necessarily increase the risk of depression in older adults with pain. Therefore, promoting caregiving benefit discovery and guiding positive pain cognition may help minimize the psychological adverse effects of physiological and social stress, such as pain and caregiving, in the lives of older adults.

## Electronic supplementary material

Below is the link to the electronic supplementary material.

Supplementary Material 1

## Data Availability

This study used public databases (the Health and Retirement Study database), which are open access and unrestricted.

## Declarations

### Statistics

The authors have checked to make sure that our submission conforms as applicable to the Journal’s statistical guidelines described here. The statistics were checked prior to submission by an expert statistician (Ou Chen, chenou@sdu.edu.cn).

### Data statement

The data has not been previously presented orally or by poster at scientific meetings.
