# Supplementary material for: The association of pain phenotype and providing caregiving with depressive symptom trajectory for older adults: a longitudinal analysis using the health and retirement study
Source: BMC Geriatr. 2025 Apr 23;25:270. doi: 10.1186/s12877-025-05926-5 (PMC12016469; doi:10.1186/s12877-025-05926-5)
Supplement: Supplementary file 1 — Supplementary Material 1 [file 12877_2025_5926_MOESM1_ESM.docx]

sTable 1. Fit statistics for latent class models from two to five classes

| N class | AIC | SA-BIC | Entroy | N1 | N2 | N3 | N4 | N5 |
| --- | --- | --- | --- | --- | --- | --- | --- | --- |
| 2 | 37498 | 37556 | 1.000 | 2742 | 5744 |  |  |  |
| 3 | 37105 | 37194 | 0.888 | 1460 | 1281 | 5744 |  |  |
| **4** | **36837** | **36957** | **0.861** | **1270** | **1472** | **595** | **5149** |  |
| 5 | 36860 | 36919 | 0.844 | 250 | 1901 | 6314 | 1276 | 594 |

SA-BIC =Sample Adjusted - Bayesian Information Criterion

sTable 2. Descriptive characteristics of participants by caregiving status

| Characteristics | Total sample | Not Providing caregiving | Providing caregiving | *F*/*χ^2^* | *P* value |
| --- | --- | --- | --- | --- | --- |
| Pain phenotype (%) |  |  |  | 411.45 | <0.001 |
| Severe persistent pain group | 1270 (15.0) | 906  (12.2) | 364  (34.7) |  |  |
| Moderate pain group | 1472 (17.3) | 1269  (17.1) | 203  (19.4) |  |  |
| Back pain group | 595  (7.0) | 517  (7.0) | 78  (7.4) |  |  |
| Pain-free group | 5149 (60.7) | 4745  (63.8) | 404  (38.5) |  |  |
| Pain persistence (%) |  |  |  | 317.57 | <0.001 |
| Pain-free | 4051  (47.7) | 3777  (50.8) | 274  (26.1) |  |  |
| Single-occurrence pain | 1730  (20.4) | 1531  (20.6) | 199  (19.0) |  |  |
| Persistent pain | 2705  (31.9) | 2129  (28.6) | 576  (54.9) |  |  |
| Pain intensity (%) |  |  |  | 361.90 | <0.001 |
| Pain-free | 5744  (67.7) | 5262  (70.8) | 482  (45.9) |  |  |
| Mild pain | 2303  (27.1) | 1891  (25.4) | 412  (39.3) |  |  |
| Moderate/severe pain | 439  (5.2) | 284  (3.8) | 155  (14.8) |  |  |
| Pain interference (%) |  |  |  | 437.37 | <0.001 |
| Pain-free | 5744  (67.7) | 5262  (70.8) | 482  (45.9) |  |  |
| No Activity-interfering pain | 1044  (12.3) | 940  (12.6) | 104  (9.9) |  |  |
| Activity-interfering pain | 1698  (20.0) | 1235  (16.6) | 463  (44.1) |  |  |
| Having back pain (%) | 3089  (36.4) | 2536  (34.1) | 553  (52.7) | 137.64 | <0.001 |

sTable 3. Linear mixed model stratified by sex, 2008-2020

| Characteristics | Males | | | Females | | |
| --- | --- | --- | --- | --- | --- | --- |
|  | Coefficient | 95% CI | *P* value | Coefficient | 95% CI | *P* value |
| Intercept | 0.967 | 0.386 to 1.547 | 0.001 | 2.061 | 1.494 to 2.629 | < 0.001 |
| Time, slope | 0.039 | 0.031 to 0.046 | < 0.001 | 0.042 | 0.036 to 0.051 | < 0.001 |
| Pain phenotype |  |  |  |  |  |  |
| Severe persistent pain group | 0.933 | 0.764 to 1.102 | < 0.001 | 1.125 | 0.987 to 1.264 | < 0.001 |
| Moderate pain group | 0.309 | 0.183 to 0.434 | < 0.001 | 0.488 | 0.365 to 0.611 | < 0.001 |
| Back pain group | 0.263 | 0.075 to 0.452 | < 0.001 | 0.446 | 0.271 to 0.621 | < 0.001 |
| Pain-free group | Ref. |  |  |  |  |  |
| Pain phenotype*time |  |  |  |  |  |  |
| Severe persistent pain group*Time | 0.003 | -0.020 to 0.026 | 0.788 | -0.024 | -0.041 to -0.007 | 0.006 |
| Moderate pain group*Time | 0.002 | -0.015 to 0.018 | 0.836 | -0.003 | -0.019 to 0.012 | 0.662 |
| Back pain group*Time | 0.014 | -0.013 to 0.041 | 0.308 | -0.007 | -0.029 to 0.015 | 0.536 |
| Pain-free group*Time | Ref. |  |  |  |  |  |
| Caregiving status |  |  |  |  |  |  |
| Providing caregiving | 0.376 | 0.146-0.606 | 0.001 | 0.483 | 0.251-0.715 | < 0.001 |
| Not providing caregiving | Ref. |  |  |  |  |  |
| Caregiving status*Time |  |  |  |  |  |  |
| Providing caregiving*Time | 0.019 | -0.021 to 0.059 | 0.359 | 0.045 | 0.007 to 0.084 | 0.021 |
| Not providing caregiving*Time | Ref. |  |  |  |  |  |
| Providing caregiving*Pain phenotype |  |  |  |  |  |  |
| Providing caregiving*Severe persistent pain group | 0.101 | -0.272 to 0.474 | 0.595 | 0.020 | -0.289 to 0.329 | 0.899 |
| Providing caregiving*Moderate pain group | 0.201 | -0.184 to 0.585 | 0.307 | -0.326 | -0.682 to 0.029 | 0.072 |
| Providing caregiving*Back pain group | -0.452 | -1.007 to 0.104 | 0.111 | 0.659 | 0.139 to 1.178 | 0.013 |
| Providing caregiving*Pain-free group | Ref. |  |  |  |  |  |
| Providing caregiving*Pain phenotype*Time |  |  |  |  |  |  |
| Providing caregiving*Severe persistent pain group*Time | -0.025 | -0.093 to 0.044 | 0.478 | -0.074 | -0.125 to -0.024 | 0.004 |
| Providing caregiving*Moderate pain group*Time | -0.003 | -0.072 to 0.066 | 0.938 | -0.052 | -0.111 to 0.007 | 0.087 |
| Providing caregiving*Back pain group*Time | -0.052 | -0.178 to 0.073 | 0.414 | -0.107 | -0.201 to -0.013 | 0.026 |
| Providing caregiving*Pain-free group*Time | Ref. |  |  |  |  |  |

sTable 4. Linear mixed model stratified by marital status, 2008-2020

| Characteristics | Married | | | Not married | | |
| --- | --- | --- | --- | --- | --- | --- |
|  | Coefficient | 95% CI | *P* value | Coefficient | 95% CI | *P* value |
| Intercept | 0.667 | 0.145 to 1.190 | 0.012 | 2.559 | 1.891 to 3.226 | < 0.001 |
| Time, slope | 0.046 | 0.039 to 0.052 | < 0.001 | 0.033 | 0.023 to 0.042 | < 0.001 |
| Pain phenotype |  |  |  |  |  |  |
| Severe persistent pain group | 0.867 | 0.736 to 0.998 | < 0.001 | 1.296 | 1.177 to 1.474 | < 0.001 |
| Moderate pain group | 0.363 | 0.259 to 0.468 | < 0.001 | 0.518 | 0.360 to 0.677 | < 0.001 |
| Back pain group | 0.334 | 0.179 to 0.489 | < 0.001 | 0.442 | 0.220 to 0.665 | < 0.001 |
| Pain-free group | Ref. |  |  |  |  |  |
| Pain phenotype*time |  |  |  |  |  |  |
| Severe persistent pain group*Time | 0.001 | -0.017 to 0.018 | 0.920 | -0.033 | -0.055 to -0.011 | 0.003 |
| Moderate pain group*Time | -0.002 | -0.015 to 0.012 | 0.797 | 0.001 | -0.019 to 0.021 | 0.933 |
| Back pain group*Time | 0.012 | -0.009 to 0.033 | 0.264 | -0.014 | -0.042 to 0.014 | 0.323 |
| Pain-free group*Time | Ref. |  |  |  |  |  |
| Caregiving status |  |  |  |  |  |  |
| Providing caregiving | 0.394 | 0.180-0.608 | < 0.001 | 0.498 | 0.238-0.758 | < 0.001 |
| Not providing caregiving | Ref. |  |  |  |  |  |
| Caregiving status*Time |  |  |  |  |  |  |
| Providing caregiving*Time | 0.044 | 0.007 to 0.080 | 0.020 | 0.029 | -0.015 to 0.073 | 0.195 |
| Not providing caregiving*Time | Ref. |  |  |  |  |  |
| Providing caregiving*Pain phenotype |  |  |  |  |  |  |
| Providing caregiving*Severe persistent pain group | 0.101 | -0.272 to 0.474 | 0.595 | 0.057 | -0.304 to 0.419 | 0.755 |
| Providing caregiving*Moderate pain group | 0.201 | -0.184 to 0.585 | 0.307 | 0.074 | -0.340 to 0.488 | 0.726 |
| Providing caregiving*Back pain group | -0.452 | -1.007 to 0.104 | 0.111 | 0.216 | -0.422 to 0.855 | 0.507 |
| Providing caregiving*Pain-free group | Ref. |  |  |  |  |  |
| Providing caregiving*Pain phenotype*Time |  |  |  |  |  |  |
| Providing caregiving*Severe persistent pain group*Time | 0.008 | -0.300 to 0.317 | 0.958 | -0.047 | -0.108 to 0.014 | 0.127 |
| Providing caregiving*Moderate pain group*Time | -0.387 | -0.726 to -0.047 | 0.026 | -0.080 | -0.155 to -0.006 | 0.035 |
| Providing caregiving*Back pain group*Time | 0.242 | -0.231 to 0.716 | 0.315 | -0.119 | -0.273 to -0.034 | 0.130 |
| Providing caregiving*Pain-free group*Time | Ref. |  |  |  |  |  |

sTable 5. Linear mixed model stratified by age groups, 2008-2020

| Characteristics | < 75 | | | ≥ 75 and < 90 | | | > 90 | | |
| --- | --- | --- | --- | --- | --- | --- | --- | --- | --- |
|  | Coefficient | 95% CI | *P* value | Coefficient | 95% CI | *P* value | Coefficient | 95% CI | *P* value |
| Intercept | 1.568 | 1.369 to 1.768 | < 0.001 | 1.416 | 1.159 to 1.674 | < 0.001 | 1.845 | 0.754 to 2.936 | 0.001 |
| Time, slope | 0.041 | 0.035 to 0.047 | < 0.001 | 0.036 | 0.027 to 0.046 | < 0.001 | 0.001 | -0.055 to 0.056 | 0.981 |
| Pain phenotype |  |  |  |  |  |  |  |  |  |
| Severe persistent pain group | 1.044 | 0.925 to 1.164 | < 0.001 | 1.159 | 0.902 to 1.217 | < 0.001 | 1.184 | 0.370 to 1.998 | 0.004 |
| Moderate pain group | 0.415 | 0.316 to 0.513 | < 0.001 | 0.419 | 0.281 to 0.556 | < 0.001 | 0.107 | -0.604 to 0.817 | 0.769 |
| Back pain group | 0.343 | 0.197 to 0.489 | < 0.001 | 0.422 | 0.226 to 0.619 | < 0.001 | 0.860 | -0.118 to 1.837 | 0.085 |
| Pain-free group | Ref. |  |  |  |  |  |  |  |  |
| Pain phenotype*time |  |  |  |  |  |  |  |  |  |
| Severe persistent pain group*Time | -0.006 | -0.022 to 0.011 | 0.508 | -0.025 | -0.047 to -0.002 | 0.034 | -0.040 | -0.175 to 0.094 | 0.558 |
| Moderate pain group*Time | -0.003 | -0.016bto 0.011 | 0.700 | 0.003 | -0.017 to 0.023 | 0.757 | -0.013 | -0.134 to 0.109 | 0.838 |
| Back pain group*Time | 0.005 | -0.015 to 0.026 | 0.615 | -0.019 | -0.048 to 0.011 | 0.213 | -0.015 | -0.168 to 0.138 | 0.851 |
| Pain-free group*Time | Ref. |  |  |  |  |  |  |  |  |
| Caregiving status |  |  |  |  |  |  |  |  |  |
| Providing caregiving | 0.353 | 0.163-0.542 | < 0.001 | 0.531 | 0.280-0.782 | < 0.001 | 0.992 | -0.445 to 2.428 | 0.176 |
| Not providing caregiving | Ref. |  |  |  |  |  |  |  |  |
| Caregiving status*Time |  |  |  |  |  |  |  |  |  |
| Providing caregiving*Time | 0.046 | 0.012 to 0.081 | 0.008 | 0.038 | -0.010 to 0.086 | 0.119 | -0.090 | -0.319 to 0.139 | 0.442 |
| Not providing caregiving*Time | Ref. |  |  |  |  |  |  |  |  |
| Providing caregiving*Pain phenotype |  |  |  |  |  |  |  |  |  |
| Providing caregiving*Severe persistent pain group | 0.021 | -0.249 to 0.292 | 0.877 | 0.121 | -0.235 to 0.477 | 0.505 | -0.916 | -2.758 to 0.926 | 0.330 |
| Providing caregiving*Moderate pain group | -0.060 | -0.365 to 0.245 | 0.699 | -0.310 | -0.709 to 0.089 | 0.128 | 0.202 | -1.863 to 2.268 | 0.848 |
| Providing caregiving*Back pain group | 0.293 | -0.140 to 0.726 | 0.184 | -0.191 | -0.795 to 0.413 | 0.536 | 0.979 | -4.783 to 6.741 | 0.739 |
| Providing caregiving*Pain-free group | Ref. |  |  |  |  |  |  |  |  |
| Providing caregiving*Pain phenotype*Time |  |  |  |  |  |  |  |  |  |
| Providing caregiving*Severe persistent pain group*Time | -0.102 | -0.150 to -0.054 | < 0.001 | -0.032 | -0.010 to 0.036 | 0.362 | 0.133 | -0.191 to 0.457 | 0.421 |
| Providing caregiving*Moderate pain group*Time | -0.063 | -0.118 to -0.007 | 0.026 | -0.009 | -0.084 to 0.067 | 0.821 | -0.013 | -0.439 to 0.412 | 0.951 |
| Providing caregiving*Back pain group*Time | -0.127 | -0.214 to -0.041 | 0.004 | 0.122 | -0.017 to 0.261 | 0.085 | 0.417 | -0.887 to 1.721 | 0.531 |
| Providing caregiving*Pain-free group*Time | Ref. |  |  |  |  |  |  |  |  |

sTable 6. Linear mixed model stratified by race, 2008-2020

| Characteristics | White | | | Black | | |
| --- | --- | --- | --- | --- | --- | --- |
|  | Coefficient | 95% CI | *P* value | Coefficient | 95% CI | *P* value |
| Intercept | 1.179 | 0.736 to 1.622 | < 0.001 | 2.799 | 1.725 to 3.874 | < 0.001 |
| Time, slope | 0.042 | 0.036 to 0.048 | < 0.001 | 0.037 | 0.0234 to 0.050 | < 0.001 |
| Pain phenotype |  |  |  |  |  |  |
| Severe persistent pain group | 1.052 | 0.938 to 1.165 | < 0.001 | 1.112 | 0.815to 1.409 | < 0.001 |
| Moderate pain group | 0.392 | 0.297 to 0.487 | < 0.001 | 0.589 | 0.334 to 0.843 | < 0.001 |
| Back pain group | 0.293 | 0.154 to 0.432 | < 0.001 | 0.818 | 0.472 to 1.164 | < 0.001 |
| Pain-free group | Ref. |  |  |  |  |  |
| Pain phenotype*time |  |  |  |  |  |  |
| Severe persistent pain group*Time | -0.013 | -0.028 to 0.002 | 0.082 | -0.031 | -0.068 to 0.005 | 0.092 |
| Moderate pain group*Time | -0.001 | -0.013 to 0.012 | 0.933 | -0.006 | -0.038 to 0.025 | 0.697 |
| Back pain group*Time | 0.007 | -0.011 to 0.026 | 0.447 | -0.031 | -0.073 to 0.0140 | 0.138 |
| Pain-free group*Time | Ref. |  |  |  |  |  |
| Caregiving status |  |  |  |  |  |  |
| Providing caregiving | 0.488 | 0.302-0.673 | < 0.001 | 0.290 | -0.082 to 0.663 | 0.127 |
| Not providing caregiving | Ref. |  |  |  |  |  |
| Caregiving status*Time |  |  |  |  |  |  |
| Providing caregiving*Time | 0.014 | -0.019to 0.048 | 0.404 | 0.086 | 0.033 to 0.139 | 0.001 |
| Not providing caregiving*Time | Ref. |  |  |  |  |  |
| Providing caregiving*Pain phenotype |  |  |  |  |  |  |
| Providing caregiving*Severe persistent pain group | -0.154 | -0.413 to 0.106 | 0.247 | 0.857 | 0.300 to 1.414 | 0.003 |
| Providing caregiving*Moderate pain group | -0.230 | -0.524 to 0.064 | 0.125 | 0.157 | -0.438 to 0.752 | 0.605 |
| Providing caregiving*Back pain group | 0.330 | -0.086 to 0.745 | 0.120 | -0.371 | -1.357 to 0.616 | 0.461 |
| Providing caregiving*Pain-free group | Ref. |  |  |  |  |  |
| Providing caregiving*Pain phenotype*Time |  |  |  |  |  |  |
| Providing caregiving*Severe persistent pain group*Time | -0.032 | -0.078 to 0.014 | 0.173 | -0.122 | -0.201 to -0.044 | 0.002 |
| Providing caregiving*Moderate pain group*Time | -0.006 | -0.057 to 0.046 | 0.825 | -0.104 | -0.200 to -0.007 | 0.036 |
| Providing caregiving*Back pain group*Time | -0.071 | -0.151 to 0.009 | 0.081 | -0.160 | -0.400 to 0.079 | 0.189 |
| Providing caregiving*Pain-free group*Time | Ref. |  |  |  |  |  |

sTable 7. Linear mixed model stratified by education levels, 2008-2020

| Characteristics | ≤ 12 years | | | > 12 years | | |
| --- | --- | --- | --- | --- | --- | --- |
|  | Coefficient | 95% CI | *P* value | Coefficient | 95% CI | *P* value |
| Intercept | 1.172 | 0.726 to 1.619 | < 0.001 | 1.137 | 0.647 to 1.627 | < 0.001 |
| Time, slope | 0.040 | 0.033 to 0.046 | < 0.001 | 0.037 | 0.028 to 0.045 | < 0.001 |
| Pain phenotype |  |  |  |  |  |  |
| Severe persistent pain group | 1.084 | 0.959 to 1.209 | < 0.001 | 1.007 | 0.866 to 1.147 | < 0.001 |
| Moderate pain group | 0.414 | 0.310 to 0.519 | < 0.001 | 0.441 | 0.324 to 0.559 | < 0.001 |
| Back pain group | 0.436 | 0.284 to 0.589 | < 0.001 | 0.280 | 0.108 to 0.452 | 0.001 |
| Pain-free group | Ref. |  |  |  |  |  |
| Pain phenotype*time |  |  |  |  |  |  |
| Severe persistent pain group*Time | -0.023 | -0.041 to -0.005 | 0.010 | 0.001 | -0.020 to 0.022 | 0.936 |
| Moderate pain group*Time | -0.003 | -0.017 to 0.011 | 0.680 | 0.003 | -0.015 to 0.020 | 0.765 |
| Back pain group*Time | -0.007 | -0.029 to 0.014 | 0.516 | 0.010 | -0.016 to 0.036 | 0.446 |
| Pain-free group*Time | Ref. |  |  |  |  |  |
| Caregiving status |  |  |  |  |  |  |
| Providing caregiving | 0.511 | 0.315 to 0.707 | < 0.001 | 0.387 | 0.159 to 0.615 | 0.001 |
| Not providing caregiving | Ref. |  |  |  |  |  |
| Caregiving status*Time |  |  |  |  |  |  |
| Providing caregiving*Time | 0.023 | -0.013 to 0.059 | 0.020 | 0.054 | 0.011 to 0.098 | 0.015 |
| Not providing caregiving*Time | Ref. |  |  |  |  |  |
| Providing caregiving*Pain phenotype |  |  |  |  |  |  |
| Providing caregiving*Severe persistent pain group | -0.070 | -0.352 to 0.211 | 0.625 | 0.209 | -0.111 to 0.528 | 0.200 |
| Providing caregiving*Moderate pain group | -0.124 | -0.436 to 0.189 | 0.437 | -0.376 | -0.741 to -0.012 | 0.043 |
| Providing caregiving*Back pain group | 0.219 | -0.244 to 0.682 | 0.354 | 0.217 | -0.316 to 0.750 | 0.426 |
| Providing caregiving*Pain-free group | Ref. |  |  |  |  |  |
| Providing caregiving*Pain phenotype*Time |  |  |  |  |  |  |
| Providing caregiving*Severe persistent pain group*Time | -0.010 | -0.061 to 0.040 | 0.688 | -0.152 | -0.213 to -0.091 | < 0.001 |
| Providing caregiving*Moderate pain group*Time | -0.021 | -0.078 to 0.036 | 0.474 | -0.029 | -0.099 to 0.042 | 0.420 |
| Providing caregiving*Back pain group*Time | -0.055 | -0.148 to 0.039 | 0.250 | -0.105 | -0.230 to 0.020 | 0.100 |
| Providing caregiving*Pain-free group*Time | Ref. |  |  |  |  |  |

sTable 8. Linear mixed model stratified by self-rated health, 2008-2020

| Characteristics | Excellent/very good/good | | | Fair/poor | | |
| --- | --- | --- | --- | --- | --- | --- |
|  | Coefficient | 95% CI | *P* value | Coefficient | 95% CI | *P* value |
| Intercept | 1.077 | 0.657 to 1.497 | < 0.001 | 3.078 | 2.104 to 4.051 | < 0.001 |
| Time, slope | 0.040 | 0.035 to 0.046 | < 0.001 | 0.047 | 0.028 to 0.066 | < 0.001 |
| Pain phenotype |  |  |  |  |  |  |
| Severe persistent pain group | 0.951 | 0.828 to 1.075 | < 0.001 | 1.184 | 0.958 to 1.409 | < 0.001 |
| Moderate pain group | 0.417 | 0.328 to 0.507 | < 0.001 | 0.475 | 0.240 to 0.710 | < 0.001 |
| Back pain group | 0.332 | 0.203 to 0.461 | < 0.001 | 0.505 | 0.166 to 0.845 | 0.004 |
| Pain-free group | Ref. |  |  |  |  |  |
| Pain phenotype*time |  |  |  |  |  |  |
| Severe persistent pain group*Time | 0.004 | -0.013 to 0.021 | 0.668 | -0.040 | -0.069 to -0.011 | 0.007 |
| Moderate pain group*Time | 0.002 | -0.010 to 0.014 | 0.731 | -0.014 | -0.045 to 0.017 | 0.387 |
| Back pain group*Time | 0.002 | -0.016 to 0.019 | 0.858 | -0.004 | -0.050 to 0.044 | 0.885 |
| Pain-free group*Time | Ref. |  |  |  |  |  |
| Caregiving status |  |  |  |  |  |  |
| Providing caregiving | 0.585 | 0.394 to 0.776 | < 0.001 | 0.311 | -0.025 to 0.646 | 0.069 |
| Not providing caregiving | Ref. |  |  |  |  |  |
| Caregiving status*Time |  |  |  |  |  |  |
| Providing caregiving*Time | 0.033 | -0.002 to 0.066 | 0.055 | 0.032 | -0.025 to 0.089 | 0.276 |
| Not providing caregiving*Time | Ref. |  |  |  |  |  |
| Providing caregiving*Pain phenotype |  |  |  |  |  |  |
| Providing caregiving*Severe persistent pain group | -0.083 | -0.433 to 0.268 | 0.644 | 0.041 | -0.376 to 0.459 | 0.846 |
| Providing caregiving*Moderate pain group | -0.122 | -0.443 to 0.200 | 0.459 | -0.184 | -0.686 to 0.319 | 0.474 |
| Providing caregiving*Back pain group | 0.373 | -0.126 to 0.871 | 0.143 | 0.070 | -0.635 to 0.775 | 0.845 |
| Providing caregiving*Pain-free group | Ref. |  |  |  |  |  |
| Providing caregiving*Pain phenotype*Time |  |  |  |  |  |  |
| Providing caregiving*Severe persistent pain group*Time | -0.014 | -0.073 to 0.046 | 0.654 | -0.057 | -0.127 to 0.014 | 0.116 |
| Providing caregiving*Moderate pain group*Time | -0.027 | -0.083 to 0.030 | 0.351 | -0.033 | -0.119 to 0.052 | 0.444 |
| Providing caregiving*Back pain group*Time | -0.164 | -0.266 to -0.061 | 0.002 | -0.029 | -0.161 to 0.102 | 0.660 |
| Providing caregiving*Pain-free group*Time | Ref. |  |  |  |  |  |

sTable 9. Linear mixed model using imputed database, 2008-2020

| Characteristics | Coefficient | 95% CI | *P* value |
| --- | --- | --- | --- |
| Intercept | 1.555 | 1.213 to 1.897 | < 0.001 |
| Time, slope | 0.035 | 0.030 to 0.039 | < 0.001 |
| Pain phenotype |  |  |  |
| Severe persistent pain group | 0.405 | 0.285 to 0.524 | < 0.001 |
| Moderate pain group | 0.413 | 0.333 to 0.494 | < 0.001 |
| Back pain group | 1.103 | 1.008 to 1.198 | < 0.001 |
| Pain-free group | Ref. |  |  |
| Pain phenotype*time |  |  |  |
| Severe persistent pain group*Time | 0.006 | -0.009 to 0.021 | 0.460 |
| Moderate pain group*Time | <0.001 | -0.010 to 0.010 | 0.955 |
| Back pain group*Time | -0.019 | -0.031 to -0.008 | 0.001 |
| Pain-free group*Time | Ref. |  |  |
| Caregiving status |  |  |  |
| Providing caregiving | 0.488 | 0.328-0.648 | < 0.001 |
| Not providing caregiving | Ref. |  |  |
| Caregiving status*Time |  |  |  |
| Providing caregiving*Time | 0.032 | 0.006 to 0.059 | 0.016 |
| Not providing caregiving*Time | Ref. |  |  |
| Providing caregiving*Pain phenotype |  |  |  |
| Providing caregiving*Severe persistent pain group | 0.092 | -0.272 to 0.456 | 0.622 |
| Providing caregiving*Moderate pain group | -0.107 | -0.356 to 0.141 | 0.398 |
| Providing caregiving*Back pain group | 0.033 | -0.184 to 0.251 | 0.764 |
| Providing caregiving*Pain-free group | Ref. |  |  |
| Providing caregiving*Pain phenotype*Time |  |  |  |
| Providing caregiving*Severe persistent pain group*Time | -0.075 | -0.141 to -0.010 | 0.025 |
| Providing caregiving*Moderate pain group*Time | -0.045 | -0.086 to -0.004 | 0.030 |
| Providing caregiving*Back pain group*Time | -0.068 | -0.103 to -0.033 | < 0.001 |
| Providing caregiving*Pain-free group*Time | Ref. |  |  |

sTable 10. Linear mixed models exploring the driving characteristics among different pain phenotypes, 2008-2020

| Characteristics | Severe persistent pain group | Moderate pain group | Back pain group | Pain-free group |
| --- | --- | --- | --- | --- |
|  | Coefficient  (95% CI) | Coefficient  (95% CI) | Coefficient  (95% CI) | Coefficient  (95% CI) |
| Pain interference (ref: pain-free) |  |  |  |  |
| No Activity-interfering pain | -0.703  (-1.468 to 0.062) | -0.265**  (-0.462 to -0.068) | NA | NA |
| Activity-interfering pain | NA | NA | NA | NA |
| Pain interference*Time |  |  |  |  |
| No Activity-interfering pain*Time | < -0.001  (-0.102 to 0.101) | 0.013  (-0.012 to 0.038) | NA | NA |
| Activity-interfering pain*Time | NA | NA | NA | NA |
| Providing caregiving*Pain interference  (ref: not roviding caregiving and pain-free) |  |  |  |  |
| Providing caregiving*No Activity-interfering pain | -1.094  (-2.977 to 0.790) | 0.016  (-0.492 to 0.524) | NA | NA |
| Providing caregiving*Activity-interfering pain | NA | NA | NA | NA |
| Providing caregiving*Pain interference*Time |  |  |  |  |
| Providing caregiving*No Activity-interfering pain*Time | 0.184  (-0.743 to 1.111) | 0.032  (-0.050 to 0.114) | NA | NA |
| Providing caregiving*Activity-interfering pain*Time | NA | NA | NA | NA |
| Pain intensity (ref: pain-free) |  |  |  |  |
| Mild pain | -0.271  (-1.399 to 0.452) | -0.406  (-0.926 to 0.114) | NA | NA |
| Moderate/severe pain | NA | NA | NA | NA |
| Pain intensity*Time |  |  |  |  |
| Mild pain*Time | 0.048*  (0.006 to 0.090) | 0.017  (-0.051 to 0.086) | NA | NA |
| Moderate/severe pain*Time | NA | NA | NA | NA |
| Providing caregiving*Pain intensity  (ref: not roviding caregiving and pain-free) |  |  |  |  |
| Providing caregiving*Mild pain | 0.283  (-0.242 to 0.809) | -0.109  (-1.336 to 1.118) | NA | NA |
| Providing caregiving*Moderate/severe pain | NA | NA | NA | NA |
| Providing caregiving*Pain intensity *Time |  |  |  |  |
| Providing caregiving*Mild pain*Time | -0.054  (-0.131 to 0.024) | 0.135  (-0.099 to 0.370) | NA | NA |
| Providing caregiving*Moderate/severe pain*Time | NA | NA | NA | NA |
| Pain persistence (ref: pain-free) |  |  |  |  |
| Single-occurrence pain | -0.385  (-1.425 to 0.655) | -0.170  (-0.357 to 0.017) | -0.346*  (-0.644 to -0.049) | 0.251***  (0.157 to 0.345) |
| Persistent pain | NA | NA | NA | 0.391***  (0.206 to 0.577) |
| Pain persistence*Time |  |  |  |  |
| Single-occurrence pain*Time | -0.143*  (-0.274 to -0.012) | -0.030*  (-0.053 to -0.007) | < -0.001  (-0.037 to 0.036) | 0.024***  (0.010 to 0.037) |
| Persistent pain*Time | NA | NA | NA | 0.022  (-0.004 to 0.049) |
| Providing caregiving*Pain persistence  (ref: not roviding caregiving and pain-free) |  |  |  |  |
| Providing caregiving*Single-occurrence pain | -1.329  (-3.710 to 1.051) | 0.270  (-0.276 to 0.817) | 0.817*  (0.006 to 1.628) | 0.167  (-0.151 to 0.484) |
| Providing caregiving*Persistent pain | NA | NA | NA | 0.672*  (0.140 to 1.204) |
| Providing caregiving*Pain persistence *Time |  |  |  |  |
| Providing caregiving*Single-occurrence pain*Time | 0.217  (-0.098 to 0.531) | -0.003  (-0.089 to 0.082) | -0.118  (-0.266 to 0.031) | 0.014  (-0.052 to 0.080) |
| Providing caregiving*Persistent pain*Time | NA | NA | NA | 0.009  (-0.101 to 0.120) |
| Back pain | 0.444  (-0.234 to 1.122) | 0.245**  (0.070 to 0.420) | NA | 0.176**  (0.071 to 0.282) |
| Back pain*Time | -0.042  (-0.135 to 0.050) | -0.003  (-0.026 to 0.019) | NA | 0.012  (-0.003 to 0.027) |
| Providing caregiving*Back pain | -0.404  (-1.570 to 0.761) | -0.103  (-0.646 to 0.440) | NA | -0.062  (-0.417 to 0.292) |
| Providing caregiving*Back pain*Time | 0.076  (-0.116 to 0.268) | 0.005  (-0.080 to 0.090) | NA | 0.040  (-0.029 to 0.108) |

CI: Credit Limit. *: <0.05, **: <0.01, ***: <0.001.

sTable 11. Linear mixed model using refined caregiving role

| Characteristics | Coefficient | 95% CI | *P* value |
| --- | --- | --- | --- |
| Intercept | 1.529 | 1.119 to 1.939 | < 0.001 |
| Time, slope | 0.041 | 0.036 to 0.046 | < 0.001 |
| Pain phenotype (ref: pain-free group) |  |  |  |
| Severe persistent pain group | 1.075 | 0.969 to 1.182 | < 0.001 |
| Moderate pain group | 0.421 | 0.332 to 0.510 | < 0.001 |
| Back pain group | 0.379 | 0.250 to 0.509 | < 0.001 |
| Pain phenotype*time |  |  |  |
| Severe persistent pain group*Time | -0.016 | -0.029 to -0.002 | 0.025 |
| Moderate pain group*Time | -0.001 | -0.013 to 0.010 | 0.838 |
| Back pain group*Time | 0.001 | -0.094 to 0.030 | 0.931 |
| Caregiving role (ref: not providing caregiving) |  |  |  |
| Providing caregiving as spouse | 0.333 | 0.097 to 0.569 | 0.006 |
| Providing caregiving as children | 0.485 | 0.224 to 0.745 | < 0.001 |
| Providing caregiving as relative/friend | 0.523 | 0.210 to 0.836 | 0.001 |
| Caregiving role*Time |  |  |  |
| Providing caregiving as spouse*Time | 0.041 | 0.001 to 0.081 | 0.045 |
| Providing caregiving as children*Time | 0.070 | 0.020 to 0.119 | 0.006 |
| Providing caregiving as relative/friend*Time | -0.032 | -0.094 to 0.030 | 0.309 |
| **Providing caregiving*Pain phenotype** |  |  |  |
| Providing caregiving as spouse*Severe persistent pain group | -0.160 | -0.493 to 0.173 | 0.346 |
| Providing caregiving as spouse*Moderate pain group | -0.358 | -0.745 to 0.029 | 0.070 |
| Providing caregiving as spouse*Back pain group | 0.252 | -0.275 to 0.779 | 0.349 |
| Providing caregiving as children*Severe persistent pain group | 0.242 | -0.144 to 0.628 | 0.219 |
| Providing caregiving as children*Moderate pain group | 0.083 | -0.345 to 0.511 | 0.703 |
| Providing caregiving as children*Back pain group | 0.311 | -0.445 to 1.066 | 0.420 |
| Providing caregiving as relative/friend*Severe persistent pain group | 0.126 | -0.302 to 0.554 | 0.564 |
| Providing caregiving as relative/friend*Moderate pain group | -0.049 | -0.577 to 0.479 | 0.856 |
| Providing caregiving as relative/friend*Back pain group | 0.183 | -0.535 to 0.902 | 0.617 |
| **Providing caregiving*Pain phenotype*Time** |  |  |  |
| Providing caregiving as spouse*Severe persistent pain group*Time | -0.034 | -0.088 to 0.021 | 0.228 |
| Providing caregiving as spouse*Moderate pain group*Time | 0.011 | -0.051 to 0.074 | 0.725 |
| Providing caregiving as spouse*Back pain group*Time | -0.060 | -0.151 to 0.031 | 0.196 |
| Providing caregiving as children*Severe persistent pain group*Time | -0.116 | -0.185 to -0.046 | 0.001 |
| Providing caregiving as children*Moderate pain group*Time | -0.082 | -0.160 to -0.004 | 0.040 |
| Providing caregiving as children*Back pain group*Time | -0.265 | -0.447 to -0.082 | 0.004 |
| Providing caregiving as relative/friend*Severe persistent pain group*Time | -0.033 | -0.113 to 0.047 | 0.422 |
| Providing caregiving as relative/friend*Moderate pain group*Time | -0.080 | -0.182 to 0.021 | 0.121 |
| Providing caregiving as relative/friend*Back pain group*Time | -0.028 | -0.215 to 0.158 | 0.764 |

CI: Credit Limit

sTable 12. Linear mixed model examining the mediating role of pain phenotype between caregiving status and depressive trajectory, 2008-2020

| Characteristics | Model1 ^a^ | | | Model2 ^b^ | | |
| --- | --- | --- | --- | --- | --- | --- |
|  | Coefficient | 95% CI | *P* value | Coefficient | 95% CI | *P* value |
| Intercept | 1.774 | 1.354 to 2.195 | < 0.001 | 1.487 | 1.079 to 1.896 | < 0.001 |
| Time, slope | 0.039 | 0.035 to 0.044 | < 0.001 | 0.039 | 0.035 to 0.044 | < 0.001 |
| Pain phenotype |  |  |  |  |  |  |
| Severe persistent pain group | NA |  |  | 1.016 | 0.927 to 1.105 | < 0.001 |
| Moderate pain group | NA |  |  | 0.394 | 0.317 to 0.472 | < 0.001 |
| Back pain group | NA |  |  | 0.390 | 0.278 to 0.502 | < 0.001 |
| Pain-free group | Ref. |  |  | Ref. |  |  |
| Caregiving status |  |  |  |  |  |  |
| Providing caregiving | 0.542 | 0.412 to 0.672 | < 0.001 | 0.467 | 0.340 to 0.594 | < 0.001 |
| Not providing caregiving | Ref. |  |  | Ref. |  |  |
| Caregiving status*Time |  |  |  |  |  |  |
| Providing caregiving*Time | -0.004 | -0.020 to 0.012 | 0.607 | -0.007 | -0.023 to 0.010 | 0.427 |
| Not providing caregiving*Time | Ref. |  |  | Ref. |  |  |

CI: Credit Limit

a: Model1 included the variable of caregiving status and its interaction with time, however, the variable of pain phenotype and theirs interaction with time were not included in this model.

b: Model2 included the variable of caregiving status and its interaction with time, as well as the variable of pain phenotype and theirs interaction with time, to test the potential cross-sectionally mediating effect of pain phenotype.
